# Regenerative Medicine Therapies for Targeting Neuroinflammation After Stroke

**DOI:** 10.3389/fneur.2018.00734

**Published:** 2018-09-03

**Authors:** Olivera Rajkovic, Geoffrey Potjewyd, Emmanuel Pinteaux

**Affiliations:** Faculty of Biology, Medicine and Health, The University of Manchester, Manchester, United Kingdom

**Keywords:** stroke, neuroinflammation, stem cells, hydrogels, nanoparticles

## Abstract

Inflammation is a major pathological event following ischemic stroke that contributes to secondary brain tissue damage leading to poor functional recovery. Following the initial ischemic insult, post-stroke inflammatory damage is driven by initiation of a central and peripheral innate immune response and disruption of the blood-brain barrier (BBB), both of which are triggered by the release of pro-inflammatory cytokines and infiltration of circulating immune cells. Stroke therapies are limited to early cerebral blood flow reperfusion, and whilst current strategies aim at targeting neurodegeneration and/or neuroinflammation, innovative research in the field of regenerative medicine aims at developing effective treatments that target both the acute and chronic phase of inflammation. Anti-inflammatory regenerative strategies include the use of nanoparticles and hydrogels, proposed as therapeutic agents and as a delivery vehicle for encapsulated therapeutic biological factors, anti-inflammatory drugs, stem cells, and gene therapies. Biomaterial strategies—through nanoparticles and hydrogels—enable the administration of treatments that can more effectively cross the BBB when injected systemically, can be injected directly into the brain, and can be 3D-bioprinted to create bespoke implants within the site of ischemic injury. In this review, these emerging regenerative and anti-inflammatory approaches will be discussed in relation to ischemic stroke, with a perspective on the future of stroke therapies.

## Introduction

Stroke is the second leading cause of death worldwide, causing 6.2 million deaths each year accounting for 12 percent of all deaths, with stroke-related illness, disability and early death set to double by 2035 (1–3). A stroke occurs due to the disruption of blood flow to the brain by a bleed (hemorrhagic stroke) (4) or a blockage (ischemic stroke), accounting for 15 and 85% of all strokes respectively. While brain tissue ischemia occurs in ischemic stroke, it remains unclear whether cerebral ischemia plays an important role during hemorrhagic stroke. In both cases however, acute insult to the brain leads to the formation of a cavity, or necrotic infarct and a cavity (5). Current therapy for ischemic stroke is limited to thrombolysis by intravenous (i.v.) administration of recombinant tissue plasminogen activator (rt-PA) given within 4.5 h of symptom onset, but is associated with unwanted effects (6), or endovascular thrombectomy to physically remove the blood clot (7). An endovascular thrombectomy can be performed as a complement to rt-PA, but like thrombolysis, it has to be carried out within hours of stroke onset and can be given to only a limited number of patients (7). Ultimately, long-term rehabilitation therapy is available to most stroke patients receiving daily sessions of motor functions, cognitive, and speech language therapies, which has proven beneficial to regain functional recovery to some extent (8).

The past decades has seen a large number of promising therapeutic approaches in pre-clinical settings, however most have failed to translate into clinical application. The reasons for these failures remain largely unknown, and the Stroke Therapy Academic Industry Roundtable (STAIR) (9) followed by STAIR meetings (10) formulated several recommendations with the hope that ongoing preclinical strategies could translate into successful therapies. One main hypothesis behind the failure of clinical trials in stroke is that current animal models are inadequate and simply do not replicate the human pathology. As a result, current therapies remain exclusively limited to thrombolysis and thrombectomy, and with an aging population and access of developing countries to western lifestyle, the clinical and socioeconomic impact of stroke and stroke-related complications is on the rise, which is further potentiated by decreased post-stroke mortality rate and patient care costs due to better rehabilitation and clinical management procedures.

Despite the aforementioned interventions, no effective treatment to promote brain tissue repair and restore brain functions after stroke exist. Regenerative medicine is an emerging paradigm in the field of stroke therapy that offers the potential to promote recovery and regeneration of damaged neurovascular tissue at previously unattainable levels. This builds on previous research into neuroinflammation intertwined with the multidisciplinary research field of regenerative medicine, utilizing biomaterials science and mechanical engineering, as well as cell and gene therapies. This review focusses on the use and limitations of anti-inflammatory regenerative medicine therapies for stroke, with specific focus on the use of nanoparticles (NPs), hydrogels, stem cells and gene-editing technologies to repair the damaged brain tissue after stroke. The use of NPs and hydrogels in particular has the potential to improve the administration of drug and cell-based therapies through a controlled release of therapeutics at appropriate doses, and therefore may enable the repurposing or revised investigation of previously ineffective therapeutics.

## Inflammation in stroke

**Figure 1:**
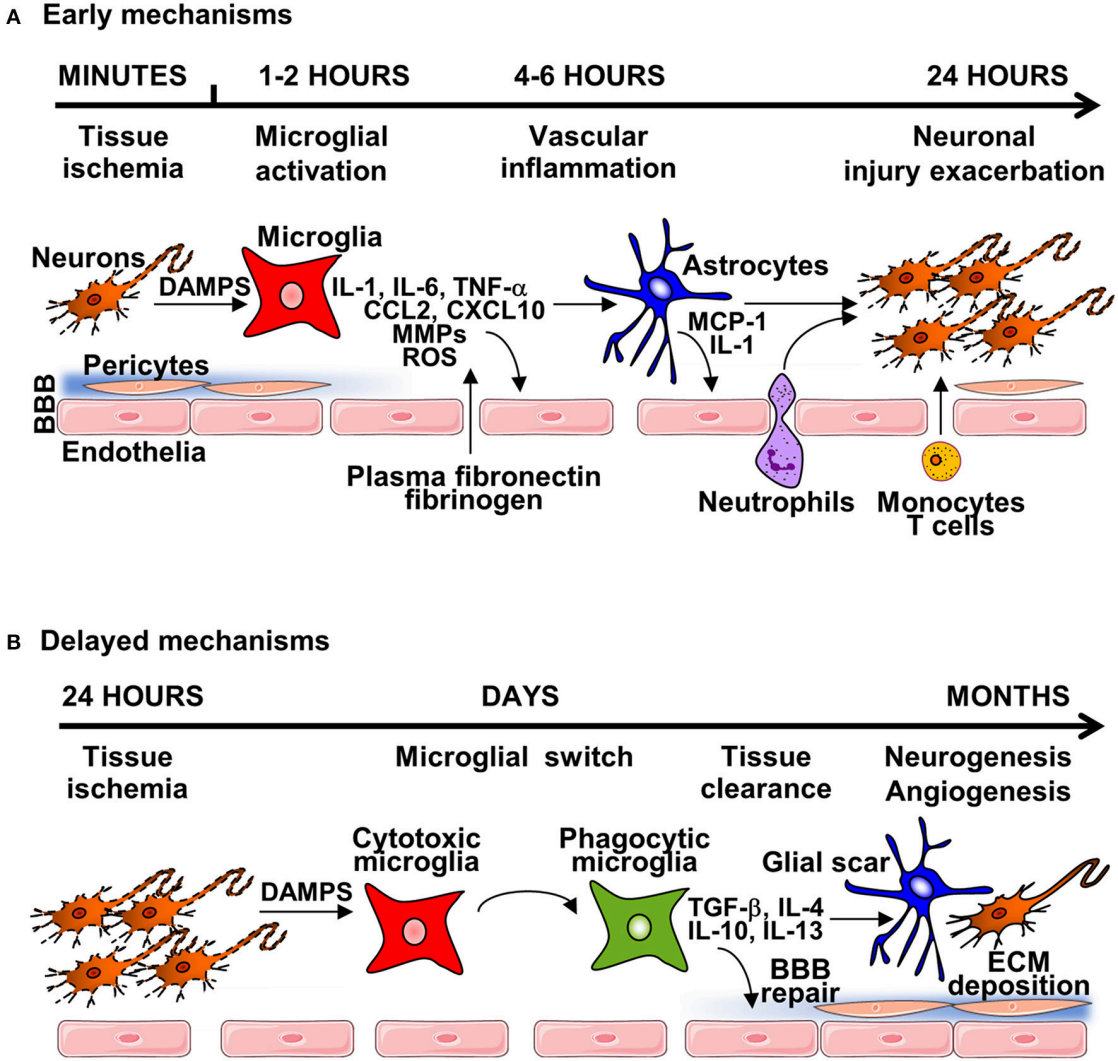
The general mechanisms of neuroinflammation post-stroke. **(A)** Early mechanisms of neuroinflammation are initiated by acute neuronal injury producing DAMPS, leading to microglial and endothelial cell activation and disruption of the BBB, through release of pro-inflammatory cytokines, chemokines, reactive oxygen species (ROS) and matrix-metalloproteinases (MMPs). Degradation of the extracellular matrix (ECM)—in both the parenchyma and basement membrane induces astrocyte endfeet and pericytes lifting from the endothelium. Damage of the BBB enables infiltration of circulatory cells with transmigration of neutrophils and immune cells. This damage can lead to brain oedema and hemorrhage, causing further neuroinflammation and tissue damage. **(B)** During the subacute phase of injury, microglial switch from cytotoxic to phagocytic phenotype occurs, leading to tissue clearance, and expression of anti-inflammatory mediators and neurotrophic factors that leads to the formation of the glial scar, and initiation of brain repair mechanisms, including neurogenesis, angiogenesis and BBB repair.

Shortly after vessel occlusion, post-ischemic inflammation begins in the vascular compartment, peaking during the first days after stroke onset (11). Post-stroke inflammation response is characterized by blood-brain barrier (BBB) disruption, infiltration of peripheral leukocytes, activation of glial cells and the release of molecules known as damage-associated molecular patterns (DAMPs) by injured and dying cells (Figure 1). Activated immune cells, triggered by DAMPs, produce inflammatory cytokines, chemokines, and other cytotoxic mediators, leading to exacerbation of cerebral ischemic injury (12). During the sub-acute phase of stroke (weeks to months after stroke onset) chronic inflammation and tissue remodeling (neurogenesis and angiogenesis) take place, although ultimately repair is limited and a fluid filled cavity develops, preventing full functional recovery (13, 14).

### Early mechanisms of neuroinflammation

Under normal conditions, microglia, the main resident immune cells in the brain, are primarily involved in monitoring (surveying) the brain parenchyma, and are known to play an important homeostatic role (15). In response to cerebral ischemia, microglia are rapidly activated, switching from a resting state to an activated state (16). The inflammatory phenotype of early activated microglia is characterized by the production of a variety of pro-inflammatory cytokines including interleukin (IL)-1, IL-6, tumor necrosis factor (TNF)-α, chemokines CCL2, and CXCL10, reactive oxygen species (ROS), nitric oxide (NO), and proteolytic enzymes such as matrix metalloproteinase (MMP)-9 and MMP-3 (17). The release of cytokines and chemokines by activated microglia/macrophage promotes recruitment of circulating immune cells to damaged brain tissue that plays a critical role in pathophysiological events following very acute stroke onset (18). Such events are associated with BBB disruption and degradation of the associated extracellular matrix (ECM), alongside activation of perivascular astrocytes. After the onset of stroke, the BBB is rapidly disrupted allowing uncontrolled entry of circulating molecules into the brain parenchyma, and this disruption persists for days through the acute and early subacute phases of stroke (19). Clinically, BBB disruption leads to the development of hemorrhagic transformation that is associated with worse stroke outcome (20), and MMPs have been identified to play a key role in this process, degrading all components of the ECM including laminin, collagen and fibronectin, and the endothelial junction proteins claudin-5, occluding, and zona occludens (ZO)-1 (21). Opening of the BBB allows penetration of plasma-derived factors (plasma fibronectin, fibrinogen) and inflammatory cells into the brain tissue, causing edema and cell death (22). Astrocytic death is a critical contributing step of BBB dysfunction in stroke by decreasing expression of tight junction proteins (23). Perivascular astrocytes express the passive water channel aquaporin 4 (AQP4) at astrocytic end-feet localized adjacent to the brain endothelium that contributes to post-stroke edema (24). In addition, astrocytes secrete chemokines such as monocyte chemoattractant protein-1 (MCP-1), a critical mediator involved in opening of the BBB after stroke (25). Astrocytes also synthesize a large array of cytokines (i.e., IL-1α, IL-1β, TNF-α) (26) that can directly trigger endothelial cell activation that contributes directly to BBB disruption (27).

### Delayed mechanisms of neuroinflammation and brain repair

Cerebral ischemia also activates important delayed endogenous repair processes such as BBB repair, neurogenesis, and angiogenesis that are important for functional recovery and patient rehabilitation in clinical settings, and evidence suggests that the sub-acute phase of inflammation plays a key role in this process. The anti-inflammatory phenotype of microglia exhibits neuroprotective and anti-inflammatory effects during the delayed phase of post-stroke inflammation, producing anti- inflammatory cytokines such as IL-10, transforming growth factor (TGF)-β, IL-4, and IL-13, as well as scavenge receptors, contributing to inhibiting inflammation and promoting tissue repair mechanisms (28). Of those, neurogenesis—known to take place in the sub granular zone of the dentate gyrus of the hippocampus and in the sub ventricular zone adjacent to the third ventricle (29)—is increased following experimental stroke (30), and is regulated by inflammatory mediators expressed during the acute phase of stroke (31). However, neurogenesis in the adult mammalian brain has been debated, with Sorrells and colleagues, reporting that human hippocampal neurogenesis declines rapidly during early childhood and is rarely detected in adult humans (32), and the role of neurogenesis on functional recovery in human remains unclear. In parallel, reactive astrocytes form a glial scar around the ischemic infarct by 14 days (33). Astrocytes initially proliferate and then migrate toward the site of ischemic injury that becomes surrounded by multiple layers of reactive astrocytes interspersed with activated microglia and a dense network of ECM proteins such as laminin, fibronectin, and chondroitin sulfate proteoglycans, resulting in the formation of a very tight glial scar (34). Angiogenesis is also a mechanism of recovery induced by inflammation after an ischemic stroke (35) that is essential for the reoxygenation of post-ischemic brain tissue, and is also an essential step for BBB repair, neurogenesis, and neuronal synaptic plasticity (11).

Although there is clinical evidence to support that inflammation plays a key role in stroke (36–39), the failure of anti-inflammatory strategies have raised hypotheses that inflammation might not play a significant role in stroke pathophysiology. This alterative hypothesis should be investigated more extensively, and future anti-inflammatory therapies may prove to be successful in the treatment of human stroke, providing more convincing evidence for the role of inflammation in stroke pathophysiology.

## Anti-inflammatory strategies in regenerative medicine

Several strategies to prevent neuroinflammation and modulate the immune response post-stroke have been studied in experimental models and explored in clinical trials (Table 1). For instance, minocycline is a semi-synthetic tetracycline derivative (51). In animal models of cerebral ischemia, minocycline administration correlates with the reduction of several pro-inflammatory cytokines, as well as ROS and NO (52). A recent comprehensive systematic review and meta-analysis by Malhotra and colleagues showed that minocycline is safe in ischemic stroke patients and demonstrated efficacy and a neuroprotective role, particularly in the acute ischemic stroke (53). Furthermore, several approaches aimed at preventing neutrophil infiltration, trafficking and/or activation have been explored; experimental models using pharmacological agents to block leukocyte adhesion and migration into the ischemic brain have shown promising results (54, 55). In particular, Fingolimod (FTY720) a sphingosine 1-phosphate receptor (S1PR) modulator that prevents the egress of lymphocytes from lymph nodes, has shown promise in preclinical models of stroke (56). This is evident in a systematic review and meta-analysis which reports that fingolimod reduced brain injury in eight out of nine studies (57). There is an ongoing Phase 2 randomized, open-label trial of patients receiving fingolimod within 72 h of ischemic stroke or spontaneous intracerebral hemorrhage. Main outcome measures include NIHSS, BI, mRS, GCS at d7, d14, d30, d90, brain MRI, and immune markers (58). In patients with small- to moderate-sized deep primary supratentorial ICH, administration of fingolimod reduced perihematomal edema, attenuated neurologic deficits, and promoted recovery. The results for ischemic stroke patients have not yet been reported. However, clinical trials testing antibodies against adhesion molecules such as intercellular cell adhesion molecule (ICAM)-1 or by administering neutrophil inhibiting factor have been inconclusive (59, 60). A recent phase 2 clinical trial, involving subcutaneous administration of interleukin-1 receptor antagonist (IL-1Ra) in ischemic stroke has shown promising results (61). IL-1Ra is known to block actions of the pro-inflammatory cytokine IL-1, which has a deleterious role in cerebral ischemia. IL-1Ra reduced plasma inflammatory markers, which are known to be associated with worse clinical outcome in ischemic stroke. Previous to this phase 2 clinical trial, recombinant human IL-1Ra (anakinra) was administered as an i.v. formulation, although it is no longer manufactured in this way (58). Anakinra was evaluated in a UK Phase 2 randomized controlled trial (RCT) in patients presenting within 6 h of acute stroke onset (39). The drug was administered intravenously and there were no significant safety concerns. Patients that received anakinra showed better clinical outcome overall and had reduced neutrophil leukocytosis, plasma C-reactive protein and plasma IL-6 levels during the 72 h infusion. Statins inhibit the enzyme 3-hydroxy-3-methylglutaryl coenzyme A reductase, lowering the level of low-density lipoprotein (LDL) cholesterol in the blood (38). In a Phase 2 RCT, patients were treated with simvastatin (40 mg/day) within 24 h after the onset of acute ischemic stroke (62). Serum TNF-α levels were marginally lower at day 3 in the simvastatin-treated group, however no clinical outcomes were reported. The safety and efficacy of simvastatin, in combination with rt-PA, is currently being evaluated in the STARS07 trial (58). Edaravone (MCI-186) is an antioxidant and free radical scavenger evaluated in a Phase 2 clinical trial for the treatment of patients with acute ischemic stroke within 24 h from the onset of symptoms (63). MCI-186 was shown to be well-tolerated and safe. However, there were no differences in clinical outcome measures after 1 year. The calcineurin inhibitor cyclosporin A has shown efficacy in preclinical stroke models. Studies reported a reduction of infarct size and inflammation as a result of the drugs suppression of cytokines, T cell activation, and ROS production (51, 64). A Phase 2 clinical study investigating the effect of cyclosporin A (single i.v. dose of cyclosporin A after i.v. thrombolysis within 4.5 h of stroke onset) on MR infarct volume at day 30 has been completed, and the results of this study has yet to be published (65).

**Table 1:** Regenerative medicine therapies for ischemic stroke.

**Therapeutic**	**Preclinical or clinical**	**Outcome**	**References**
Polyethylene glycol-melanin nanoparticles (PEG-MeNPs)	Preclinical; rat model with middle cerebral artery occlusion (MCAO)	Pre-injection of PEG-MeNPs significantly reduced infarct volume and decreased superoxide levels in brain tissues; *in vitro*, NPs decreased expression of pro-inflammatory cytokines	(40)
Carbon NPs (hydrophilic carbon clusters conjugated to PEG) termed PEG-HCCs	Preclinical; transient MCAO in acutely hyperglycemic rats	Reduction in infarct size, hemisphere swelling, hemorrhage score, and improvement in Bederson score	(41)
Perflutren lipid microspheres trademarked as Definity (Lantheus Medical Imaging)	Clinical; FDA approved (2001) ultrasound contrast agent	Safety/efficacy study for use as an ultrasound enhancer for acute ischemic stroke	(42)
Microporous Annealing Particle (MAP) hydrogels	Preclinical; mouse model with MCAO	Injection of MAP hydrogels in the stroke cavity reduces gliosis and inflammation and promotes neural progenitor cell migration to the lesion	(43)
Hyaluronic acid hydrogel mixed with poly(lactic-co-glycolic acid) microspheres (HA–PLGA) containing vascular endothelial growth factor (VEGF) and angiopoietin-1 (Ang1)	Preclinical; mouse model with MCAO	Inhibition of brain inflammation and gliosis after implantation in brain, behavior improvement recorded by cylinder testing and enhanced angiogenesis	(44)
HA gel + heparin nanoparticles (nH) with VEGF binding	Preclinical; mouse model with distal MCAO	HA gel + nH injection into the stroke cavity reduced inflammation (activated microglia and reactive astrocytes) and significantly increased vascularization within the stroke cavity and the peri-infarct area	(45)
Hydrophobic (HP) carbon nanotubes (CNTs) impregnated with subventricular zone neural progenitor cells (SVZ NPCs)	Preclinical; rat model of transient MCAO	HP CNT-SVZ NPC transplants reduced infarct cyst volume and infarct cyst area. Improved rat behavior and stem cell differentiation. Reduced inflammation (activated microglia)	(46)
Amine-modified single-walled carbon nanotubes (a-SWNTs)	Preclinical; rat model of MCAO	Injection of the right lateral ventricles 1 week before induction of ischemic stroke reduced stroke infarct volume, apoptotic, angiogenic and inflammation markers. Behavioral recovery evaluated by the Rota-Rod treadmill test	(47)
Multipotent adult progenitor cells (MAPCs) trademarked as MultiStem	Clinical: MultiStem phase II clinical trial; treatment of patients with acute ischemic stroke	Intravenous MultiStem treatment was safe and well tolerated. Lower rates of life-threatening adverse events or death and of secondary infections. Reduced biomarkers of post-stroke inflammation	(48)
CTX stem cell therapy (neural stem cell line)	Clinical; phase II clinical trial (PISCES II) for patients with motor disability as a result of ischemic stroke	Treatment was well tolerated and patients showed clinically relevant improvements in the Action Research Arm Test (ARAT) scores, Modified Rankin Scale and Barthel Index	(49)
Gene therapy; Interleukin-1 receptor antagonist (IL-1Ra)-producing bone marrow (BM) cells	Preclinical; mouse model of permanent or transient MCAO	Therapeutic injection of IL-1Ra-producing BM cells post-stroke amplified microglial production of IL-1Ra and reduced brain levels of IL-1β, collectively leading to smaller infarcts and improved functional outcome	(50)

Past and current anti-inflammatory therapies have not translated into a successful clinical treatment for ischemic stroke. Regenerative medicine therapies for stroke may alleviate some of these challenges by providing a structural support, localizing therapy to the site of action, and/or modulating endogenous regenerative cues to brain cells. The multidisciplinary nature of the regenerative medicine approach improves the likelihood of the development of an effective therapy for ischemic stroke. When considering the aforementioned therapies, cyclosporin A, edaravone, and IL-1Ra are the best candidate drugs for combination with NP delivery as they are administered intravenously, thus encapsulation into NPs would potentially improve blood circulation half-life and allow for a more targeted and controlled drug delivery. NPs could be used for the targeted therapeutic delivery of rt-PA, and Tadayon; colleagues have studied the potential of silica-coated magnetic NPs as nanocarriers for rtPA, showing promising results (66). The CTX stem cell therapy developed by ReNeuron could be encapsulated into a hydrogel, for injection into the ischemic brain, enabling controlled delivery over time and better cell survival.

Demonstrating the efficacy of emerging regenerative medicine therapies for ischemic stroke is important and challenging. Magnetic resonance imaging (MRI) can be used to visualize and quantify the infarct volume at multiple time points after stroke (67). This method can be applied to both animal models and stroke patients, and is arguably far more accurate than the determination of infarct volume by immunohistostaining such as NeuN immunostaining or cresyl violet staining (68, 69), which cannot be achieved in humans. Stroke cavity size can also be determined in order to evaluate whether a therapy is promoting repair after stroke injury. For example, Wang and colleagues used cresyl violet staining to quantify the cavity size in a mouse model of stroke (70). As demonstrated by Zhang and colleagues, the effect of scaffold implantation on the integrity of brain shape can be simply shown by haematoxylin and eosin staining of rat brain sections or by extracting and visually observing the whole brain (71). Regenerative medicine therapies may increase post-stroke neurogenesis (72), which can be assessed by doublecortin and NeuN/BrdU immunohistochemistry (73). Induction of post-stroke angiogenesis is considered to be beneficial and can be imaged by laminin immunohistochemistry (44). Immunohistochemical staining for reactive astrocytes and activated microglia is commonly used to determine whether a regenerative medicine therapy is attenuating the inflammatory response after experimental stroke (72). The translocator 18 kDa protein (TSPO) has been used in PET imaging studies to image glial activation and neuroinflammation (74). Recently, improved radioligands for this protein have been developed and approved for human imaging including (^11^C)PBR28, (^18^F)DPA-714, and (^18^F)FEPPA (75). Ultra-small superparamagnetic particles of iron oxide (USPIO) can be used for human imaging of monocyte/macrophage tracking, and have been used successfully to study neuroinflammation in stroke patients (76). The PET ligand ^11^C-flumazenil (FMZ), which targets GABA-A receptors, has been used for imaging neuronal integrity in human stroke, with patients showing reduced FMZ binding potential in ischemic brain regions (77). Tracking of transplanted stem cells is essential to monitor safety and efficiency of cell-based therapies. Citrate-coated superparamagnetic iron oxide NPs have been used for *in vivo* stem cell tracking by MRI (78). Zhu and colleagues reported a case of labeling human neural stem cells (NSC) with superparamagnetic iron oxide NPs and tracking their survival, migration, and distribution in a patient with brain trauma by MRI (79). Additional promising imaging modalities for tracking stem cells include nuclear imaging [Positron emission tomography (PET) and Single-photon emission computed tomography (SPECT)] and optical imaging (80). To evaluate changes in neurological function, animals can be subjected to a variety of somatosensory, motor, learning, and memory tests before and after surgery. For example, the rotarod test is widely used for evaluating motor function and balance in rats and mice (81). In addition, the pole test and wire hanging test can be used to assess motor dysfunction after stroke (82). Cognitive deficits including memory problems occur in human stroke survivors thus memory tests have been developed for use in animals such as water maze and passive avoidance task (83). Tests that assess anxiety-like behavior in rodent models have been developed, in order to address post-stroke anxiety that affects up to 40% of stroke survivors (84). Popular tests for this include dark-light box, Vogel conflict test, Geller- Seifter conflict test, elevated plus maze, and open field (81).

### Anti-inflammatory properties of nanoparticles (NPs)

Nanoparticles (NPs) are colloidal carriers that can be of natural or synthetic origin and can vary in size from 1 to 1,000 nm (85). Natural NPs are primarily composed of molecules such as proteins (albumin), polysaccharides, or chitosan for instance (86). Synthetic NPs are made from common polymers such as poly(lactic-co-glycolic acid) (PLGA), poly(ethylenimine) (PEI), polyesters poly(lactic acid) (PLA), or from inorganic agents such as gold, silica or alumina (87). NPs can be spherical, cubic and rod-like in shape, and they can have negative, zwitterionic, or positive charge, affecting interactions with biological substrates and the BBB (85). NPs can be coated and functionalized with different types of ligands; some are capable of mediating protein adsorption, others are able to interact directly with the BBB, increase hydrophobicity, or are able to improve blood circulation (88). NPs are versatile drug delivery systems that can be used for the targeted delivery of therapeutic agents into normally inaccessible organs like the brain, and can also be used for the delivery of lyophobic drugs (89).

Recently, it has been suggested that NPs could exert potent anti-inflammatory effects by acting on ROS production, a key process in stroke pathogenesis, since oxidative stress contributes to the initiation of the post-ischemic inflammatory response (90). Recent work from Liu et al. (40) has shown that polyethylene glycol-melanin NPs (PEG-MeNPs) exhibit broad anti-oxidative properties against multiple toxic reactive oxygen and nitrogen species (RONS) including superoxide ions (O_2_•−), hydrogen peroxide (H_2_O_2_), hydroxyl radical (•OH), peroxinitrite (ONOO–), and NO, highlighting their potential as a robust RONS scavenger (40). Using a rat model of ischemic stroke, they showed that pre-injection of PEG-MeNPs can significantly decrease ischemic brain injury. *In vitro*, the NPs were shown to be anti-inflammatory, decreasing the expression of cyclo-oxygenase 2 (COX-2), inducible nitric oxide synthase (iNOS), TNF-α, and IL-1β in lipopolysaccharides(LPS)-stimulated macrophages. Biocompatibility was assessed *in vitro* and *in vivo*, with NPs demonstrating no obvious toxicity. Another type of NPs, retinoic acid-loaded polymeric NPs (RA-NP) have been developed to modulate microglial response toward an anti-inflammatory and somehow neuroprotective phenotype (28). RA-NP were internalized by murine N9 microglial cell line and inhibited LPS-induced iNOS expression and NO release, whilst promoting arginase-1 and IL-4 production. Additionally, RA-NP effects on microglial phenotype, promoted tissue viability and neuronal survival in organotypic hippocampal slice cultures exposed to an inflammatory stimulus. A new class of antioxidant NPs composed with hydrophilic carbon clusters conjugated to poly(ethylene glycol), named PEG-HCCs, have been recently developed (41). They are effective at scavenging hydroxyl radical and have been found to reduce infarct size when administered during the reperfusion period after experimentally-induced stroke in rat (41).

Over the last decade, a variety of NPs (metal-based, carbon-based, polymer-based, biological-based, and lipid-based) have been investigated for their use in biomedical imaging (91). In particular, the potential uses of iron oxide NPs as MRI contrast agents has been an area of intense interest, and several types of these particles, such as ferumoxytol, have been approved by the Food and Drug Administration (USA) for their use in clinical diagnosis (92). Europium-doped very small iron oxide NPs have been used to visualize neuroinflammation with MRI combined with fluorescence microscopy (93). In addition, there have been recent developments in molecular imaging techniques using organic NPs and quantum dot applications for visualizing *in vivo* molecular pathways (94, 95). Clinically approved NPs are currently limited to SPECT imaging of peripheral organs such as gastrointestinal tract, liver, and spleen (96). Although nanotechnology has relieved many problems in biomedical imaging, the clinical translation of many types of NPs is impeded by fundamental limitations of human physiology (i.e., vessel pore size, renal, and hepatic clearance), potential toxicity, and/or interference with other medical tests. Hence, a refined NP design and extensive toxicity studies will help facilitate the clinical translation of new NPs that have unique advantages over conventional imaging agents.

### Anti-inflammatory properties of hydrogels

Hydrogels are acellular polymeric networks that replicate the intrinsic properties of the native ECM of the neurovascular unit (NVU) (97, 98), and are therefore used commonly for *in vitro* cell culture and as an *in vivo* therapeutic tool. The polymeric constituents of hydrogels are termed as biopolymers, which are of natural or synthetic origin (97–99). Natural hydrogels are formed of protein and polysaccharide biopolymers that are either native constituents of the ECM, i.e., collagen, laminin or hyaluronic acid, or can be structurally similar to the native ECM, like alginate and gellan gum. Synthetic hydrogels are chemically synthesized biopolymers—commonly peptide based—that can be designed to assemble into an ECM-like conformation (also known as self-assembling peptides). Hydrogels provide a supportive 3D microenvironment that is similar to the native ECM. This enables the encapsulation of cells, drugs or growth factors for injection or implantation into the brain.

**Figure 2:**
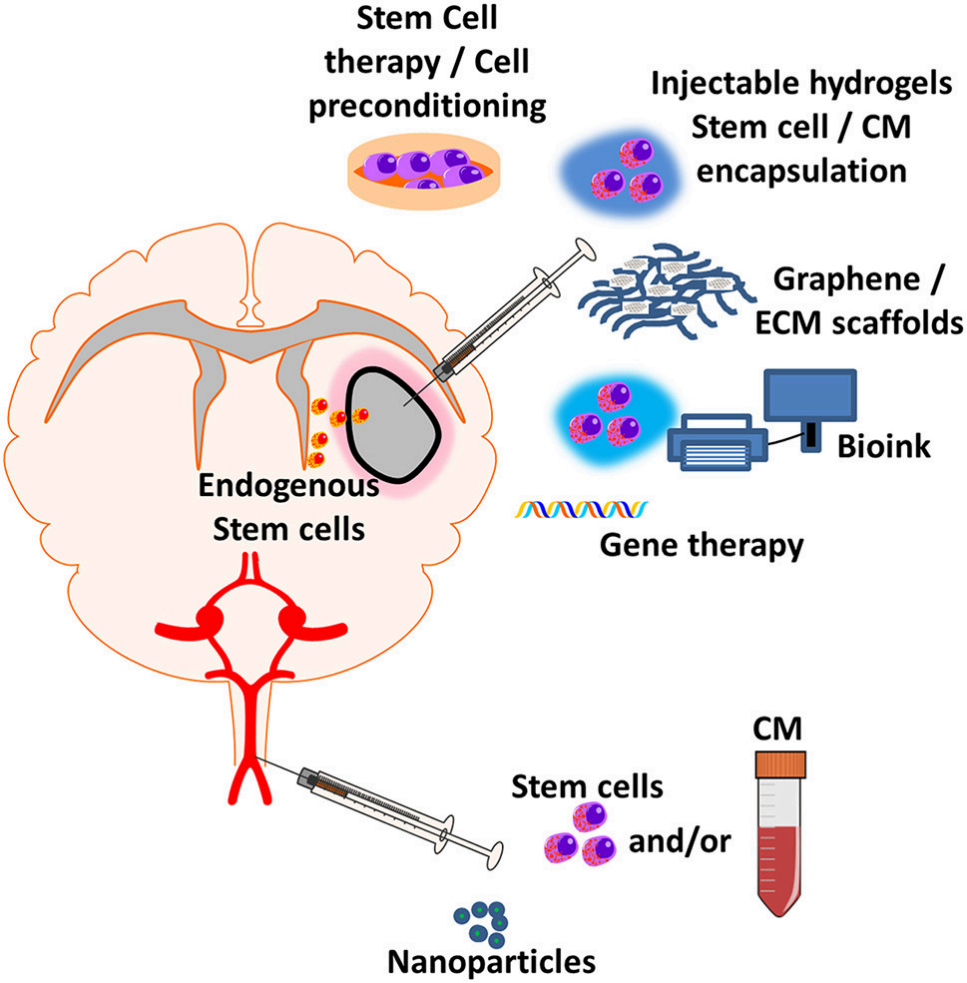
Regenerative medicine applications for treating post-stroke inflammation. New emerging regenerative medicine approaches include central injection of cell therapy and/or encapsulated factor loaded hydrogels, graphene and ECM scaffolds, 3D-bioprinting of cell therapy and/or encapsulated factor loaded bioink, gene therapy which can be implanted directly into the site of injury. Systemic injection of nanoparticles with encapsulated anti-inflammatory factor, nanoparticles or cell therapy can either elicit an effect at the blood-brain barrier (BBB) or enter the parenchyma to elicit an effect at the site of injury. Exogenous administration of new regenerative medicine therapies could lead to the recruitment and infiltration of endogenous stem cells to stroke site.

The primary aim of using hydrogels in stroke recovery is to provide an exogenous ECM-based network that allows structural support within the cerebrospinal fluid-filled cavity and promotes endogenous brain tissue repair around the ischemic lesion. To this end, hydrogels must have appropriate properties for the brain tissue, with the neurovascular environment having different ECM properties compared to other peripheral organs. Furthermore, hydrogels must be biocompatible, without activating an immune response from the native tissue, whilst promoting anti-inflammatory activity and recovery. Key physical parameters for hydrogel biocompatibility include porosity, stiffness, and preferentially the physico-chemical presence of cell adhesion peptide (CAP) domains (72, 97, 100, 101). Hydrogel porosity enables the diffusion of nutrients throughout the 3D structure. If the pore size of a hydrogel is too low then nutrients and oxygen within media may not efficiently permeate through the entire structure, or could cause a concentration gradient; potentially leading to necrotic regions (102, 103). The stiffness of a hydrogel regulates the phenotype of cells, with mechanical interactions between cells of the NVU and the ECM through hydrostatic pressures (104) and CAP binding (105, 106), even directing the differentiation of stem cells (105–108). Crosslinking is the mechanism by which a pre-gelation hydrogel becomes solid, with the initiation of inter-molecular physical or chemical bonds maintaining a 3D structure. Therefore, crosslinking dictates the administration technique used for delivering hydrogel to the site of injury, with injectable hydrogels requiring crosslinking (gelation) to occur under physiological conditions [Figure 2; (109, 110)], whereas implanted hydrogels can be crosslinked in a controlled *in vitro* situation. Hydrogel injection has been achieved with both synthetic (43, 111, 112) and natural biopolymer hydrogels (109, 113–115) for stroke and other CNS applications.

The interactions between implanted hydrogel and endogenous brain cells have the potential to induce many different reparative and anti-inflammatory cellular pathways, through binding of CAPs (including RGD, IKVAV, and YISGR motifs) to specific cell surface receptors (101). Anti-inflammatory targets of CAPs include; cell adhesion molecules (CAMs), which are involved in the recruitment and trafficking of leukocytes (51, 116); integrin receptors, which in addition to having anti-inflammatory effects can have proangiogenic properties (115, 117, 118), reduce reactive gliosis (43, 119) and promote the infiltration of neural progenitor cells to the site of injury (43, 111); and growth factor receptors that can initiate similar anti-inflammatory effects (120). CAPs can also be used to mimic growth factors and initiate preferential cellular pathways. For example a peptide (QK) which binds to the vascular endothelial growth factor (VEGF) receptor has been incorporated into a hydrogel to promote angiogenesis and may also have an anti-inflammatory effect similar to that observed after recombinant VEGF administration (120). Hydrogels like this VEGF-mimetic structure could aid recovery and promote anti-inflammatory processes following stroke, and the technique used here could be implemented for a number of growth factor mimetic peptides to promote the desired anti-inflammatory actions.

Tissues can be decellularised to isolate the native ECM, which has been used to create hydrogels with anti-inflammatory effects, whilst also aiding clearance of necrotic debris and providing a platform for regeneration through infiltration of endogenous cells to the stroke site (100, 121). Isolation of single ECM components for hydrogels enables the determination of the positive or negative effects of different biopolymers on brain tissue, with some native ECM biopolymers inducing an anti-inflammatory response on their own; Hyaluronic acid (HA) hydrogels in particular have been used frequently in stroke studies (43, 115, 122–126), owing to their anti-inflammatory effects through CAPs binding to CD44, which inhibits inflammation (127) as well as leukocyte rolling and extravasation through the BBB to the brain parenchyma (128). Similarly, gelatin has been shown to exhibit native anti-inflammatory effects in the brain following injury through repairing the BBB, reducing circulatory molecules and cells from entering the brain parenchyma and shifting the microglial response from neurotoxic to a neuroreparative phenotype (129).

Implantation of hydrogels into the brain would require invasive surgery and therefore is a higher risk regenerative strategy than hydrogel injection and other therapeutic techniques, but does offer certain advantages. Through use of 3D-bioprinting, a printable hydrogel (bioink) and brain scans, an implantable structure can be created with patient specific dimensions (Figure 2). Anti-inflammatory and patient specific bioinks can be created with use of a patient platelet-rich plasma (PRP)—a platelet rich fraction of blood that contains a number of growth factors—allowing for printed 3D-anti-inflammatory structures to be implanted (130). Certain hydrogels and bioinks require potentially toxic reagents or ultraviolet-light to initiate crosslinking through the creation of ROS, potentially damaging the cellular contents or initiating downstream pro-inflammatory pathways (131). This further highlights the need for appropriate selection of hydrogel to ensure that anti-inflammatory effects are not negated by the production procedure.

### Hydrogels for delivery of anti-inflammatory therapeutic agents

Hydrogels can also be used as a vehicle for the delivery of drugs, growth factors, stem cells, and NPs, to control delivery of therapeutics over time in conjunction with the rate of hydrogel degradation. Hydrogel degradation and gradual release of therapeutics can be tuned to the release profile desired by modifying the physical properties of the biopolymer, or by simply selecting a hydrogel with the appropriate physical profile. Whilst there has been a level of success with anti-inflammatory drugs for post-stroke recovery, the therapeutic window and dosing strategies of these drugs could be enhanced by encapsulation and controlled release from a hydrogel or NP structure (Figure 2). The half-life of drugs injected without a controlled release system is limited, whereas when administered within a hydrogel or NP, the drug can be present at the site of stroke damage for days or even weeks (70, 72, 125, 132). This is a concept which can be applied to various neurodegenerative diseases and to repairing the nervous system, justifying the re-investigation of previously promising drugs and drug targets in a hydrogel- or NP-based administration system (133, 134). Controlled release has also been used in regenerative cardiology, where the use of a hydrogel-based oxygen release system provided a sustained release of oxygen to cardiac tissue in a model of heart failure for up to 4 weeks, significantly reducing inflammation, ROS production and promoting functional recovery of the damaged tissue (135). This system could be applied to treating ischemic regions following stroke and could allow sustained release of oxygen to promote tissue recovery and regeneration. Hydrogels also allow for the controlled release of NPs into the surrounding stroked tissue, for the controlled release of anti-inflammatory NPs (45) delivering encapsulated anti-inflammatory drugs to the site of injury (44, 125).

Conditioned media are commonly produced in *in vitro* research, with astrocyte-derived conditioned media known to improve the survival and function of other cells of the NVU (136–138). The potential benefit of using cell-derived conditioned media (without cells) is to implant cell secretomes without inducing an immunogenic response from the host tissue. This also presents the opportunity to prime cells to secrete beneficial factors that can reduce inflammation and promote neurorepair in the post-stroke brain (139). Recent research has shown alterations in the secretome of mesenchymal (stromal) stem cells (MSCs) following priming with IL-1, which promotes the secretion of anti-inflammatory and proangiogenic growth factors that could aid recovery (140, 141). Similarly, encapsulation of pro-angiogenic fibroblast growth factor (FGF)-2 within a collagen-alginate hydrogel controlled release system has been shown to be beneficial to ischemic tissues in zebrafish models (142). By using hydrogel controlled release systems, it is possible to therapeutically release anti-inflammatory secretomes to aid regeneration of damaged brain tissue.

### Carbon based substrates

The integration of carbon-based substrates to the brain and in hydrogels has been investigated previously for neural tissue engineering. Two of the most commonly investigated carbon substrates for potential stroke therapy are carbon nanotubes (CNTs) and graphene, which have conductive properties that promote neurons and NSC activity and survival. CNTs have been used previously for neural tissue engineering due to their strong conductive properties, which can promote the differentiation and function of neurons (143, 144). CNTs, used as a substrate within hydrogels, have been used to promote both the expression of neural phenotypes and to secrete neurotrophic factors that could reduce inflammation (145). The transplantation of CNTs directly into the post-stroke brain has been shown to reduce microglial activation in the weeks following stroke, as well as promoting neural progenitor cell differentiation to functioning neurons (46). The administration of CNTs before stroke also exhibited enhanced recovery following stroke, with a reduced level of inflammatory markers (47).

Graphene is a biomaterial consisting of carbon in a 2D plane, like the 3D structure of graphite, but with only a single-atom thickness. For biomedical applications, graphene is commonly oxidized (graphene oxide, GO) to make the material hydrophilic and to improve biocompatibility (146, 147). The structural advantage of using GO over the 3D counterpart (graphite oxide for instance) is the enhanced surface area and hydrophilicity that is gained from having atom-thick layers (146). GO can be integrated into hydrogels—as a substrate or graphene foams—for implantation after stroke, due to its mechanical, physical, and electrical properties (148–150). GO has been shown to have ROS scavenging and immune modulating properties when conjugated with a synthetic hydrogel and injected into the post-myocardial infarction heart (151), as well as reducing neuroinflammation in a poly-ε-caprolactone scaffold through inhibition of reactive gliosis and subsequent reduction in glial scarring (148). This positive immunomodulatory response shows promise for the use of GO in hydrogel systems for stroke. More research in graphene derivatives is needed to determine toxicity and immunogenic responses when introduced into living systems—especially for prolonged periods of time—before translation to humans can be considered (152).

### Stem cell therapies

Stem cell therapy is a promising therapeutic approach in stroke and is a research priority (153). Stem cells can differentiate into many cell types including neuronal and endothelial lineage, and it has been widely assumed that once implanted they may promote recovery by repopulating the necrotic cavity present within the area of ischemic damage (154). Indeed, several studies have tested the effect of embryonic-derived NSC, induced pluripotent stem cells (iPSCs), embryonic stem cells (ESCs), MSCs, and bone marrow stem cells (BMSCs) in pre-clinical stroke models (155). Further, the world's first fully-regulated open-label clinical trial of neural stem cell (NSC) therapy in stroke (Pilot Investigation of Stem Cells in Stroke, PISCES I, ReNeuron, UK), followed by the current Phase II trial (PISCES-II) appeared safe with suggestion of functional improvement (49), whilst autologous transplantations of MSCs in stroke patients appear safe and are associated with clinical improvement (156). Although it has been long assumed that cell replacement is the primary mechanism of action of implanted stem cells, a new paradigm of stem cell actions has recently focused on their paracrine actions. It is known that MSCs for instance exert unique therapeutic effects by secreting anti-inflammatory and trophic factors that can transform the local inflammatory environment when implanted locally (157), and the anti-inflammatory theory has been established for other types of stem cell (158). To induce anti-inflammatory mechanisms, stem cells can be manipulated or genetically edited to express certain proteins that are neuroprotective and anti-inflammatory.

A type of anti-inflammatory cell therapy is the transplantation of stem cells that activate downstream cellular pathways and promote infiltration of endogenous NSC to the site of stroke injury (Figure 2). This involves the transplantation of stem cells which have either been differentiated from iPSCs, ESCs, MSCs, or BMSCs to a neural progenitor state, or are un-differentiated. The delivery of neural progenitor cells to the site of injury triggers recovery through reducing inflammation and reactive gliosis as well as promoting angiogenesis (159). The transplantation of un-differentiated pluripotent stem cells (iPSCs and ESCs) has a heightened risk of teratomas and is therefore investigated to a lesser extent (160, 161). In contrast, BMSCs and MSCs have been shown to have beneficial anti-inflammatory effects through inhibition of microglia activation without the heightened risk of tumorigenesis (162). Further research is needed to try and optimize the transplantation of pluripotent stem cells to avoid tumorigenic complications, with the transplantation of cells within a hydrogel of growth factors to direct differentiation potentially offering a better therapeutic approach.

Cell therapies are commonly administered through i.v. injection, requiring cells to cross the BBB. The selective permeability of the brain endothelium restricts cell infiltration resulting in much larger doses of cell therapy being needed to have a therapeutic effect (163, 164). To circumvent this limitation, dual therapies including stem cells administered with biomaterial, astrocyte-derived conditioned medium or drugs that transiently open the BBB have been considered (163, 165). Alternatively, therapies based on administration of T-cell (Treg), known not to cross the BBB, are able to dampen the immune response in the brain and subsequently exert anti-inflammatory actions after stroke (166–169). The anti-inflammatory and neuroprotective effect of Tregs occurs through C-C Chemokine Receptor Type 5 (CCR5) interaction with the endothelial vessel wall, which allows the Tregs to interact with circulatory macrophages and neutrophils (167). This information suggests CCR5 as a potential therapeutic target for enhancing the therapeutic effect of Tregs as well as a sole target without Treg therapy.

Studies have reported modest recovery and highlighted the need to develop new strategies to improve the safety and efficacy of stem cell therapies in stroke. *In vitro* pre-treatment of stem cells by specific culture conditions and/or biological agents (also known as “preconditioning” or “priming”) can improve the survival, engraftment, immunosuppressive and paracrine properties of stem cells, therefore enhancing their regenerative capacity. For MSCs, preconditioning strategies have been explored in order to enhance the anti-inflammatory properties of MSCs, including exposure to hypoxia/growth factors (170) and inflammatory cytokines (171), whilst the only preconditioning strategy in human stroke patients (STARTING-2) tested the transplantation of autologous MSCs exposed to autologous serum obtained at stroke onset (172). Further, the encapsulation of cells within a hydrogel can create a pre-made tissue to help promote brain repair following stroke. This approach also improves the rate of stem cell survival from implantation, as the cells have a support matrix to aid their integration in the host tissue. This has been shown through using a HA based hydrogel with growth factors, cell adhesion domains (RGD, IKVAV, and YISGR) and neural stem cells, which enhanced stem cell survival following injection in stroked mice (122). A similar HA has been used to inject NSC and subsequently differentiate to a neuronal lineage (123).

### Gene therapies

The mass advancements of gene-editing technologies has enhanced the capabilities of both cell and gene therapies, with beneficial genes being introduced to cells *in vitro* or *in vivo* to promote the expression of neuroprotective or anti-inflammatory factors. These advancements also raise ethical considerations as editing of the germ line coding sequences results in permanent and hereditary genetic changes, as opposed to editing non-germ line genes. In addition to ethical considerations it is important to ensure that editing a certain gene does not have off-target effects that could cause adverse events in patients.

The use of gene therapies offers the potential to alter cellular and molecular processes that are important to recovery from ischemic stroke, reducing the inflammatory response and initiating regeneration of damaged tissue. This approach has been used to deliver anti-inflammatory gene therapies that promote production of VEGF (116, 173), anti-inflammatory neural cell adhesion molecule (NCAM) (116), or IL-1Ra (50). These therapies were administered in rodents by intrathecal injection, but could be improved through encapsulation within a hydrogel for injection or implantation as this would control the release of gene-edited cells over time to increase the therapeutic effect.

Hydrogel- and NPs-based delivery systems enable the optimization of cell and gene therapy delivery to the site of stroke injury. Systemically injected NPs can optimize BBB permeability through precise surface chemistry and can be designed for controlled release of encapsulated cells. While hydrogels must be injected directly to the brain, they provide ECM mimetic support for both the encapsulated cells and the surrounding host tissue. Like with NPs, the degradation profile of the hydrogel biomaterial can enable the controlled release of cells to the brain; both prolonging the application of anti-inflammatory factors over time rather than having a short therapeutic effect.

Targeting genes that affect neuroinflammation has the potential to be used as an effective therapy for multiple different neurological diseases, with many of these diseases having an inflammatory pathophysiology implicated in either disease onset or progression (174, 175). As an example, pre-clinical Alzheimer's disease research has identified anti-inflammatory mediators that could be targeted using gene therapies to modulate disease pathology (176–178). In a mouse model of Alzheimer's disease, viral vectors have been used to increase gene expression of anti-inflammatory cytokines IL-2 (178) and IL-10 (177) which had a positive effect on pathology and cognitive function in mice. Similar approaches to inhibit neuroinflammation have been applied to other neurological conditions, with a multiple sclerosis gene therapy showing neuroprotective and even disease reversing clinical outcomes in a mouse model (179). The principles of gene-editing that have been developed in these neurological disease models has the potential to influence stroke gene therapy progression, with shared inflammatory pathways in stroke allowing for similar treatments to aid tissue regeneration in the post-stroke brain.

## Concluding remarks

Regenerative medicine is an emerging field of interdisciplinary research, providing potential future solutions for the treatment of stroke and other neuroinflammatory conditions. The efficacy of the regenerative approaches discussed in this review has been explored mainly in pre-clinical models showing reductions in inflammatory responses and improved recovery of brain tissue. These pre-clinical studies form the basis of scientific evidence to progress the translation of regenerative therapies toward clinical applications. In particular, the use of biomaterials as anti-inflammatory agents—or as vehicles for controlled release of anti-inflammatory agents—in the form of NPs or hydrogels present as attractive candidates for improving the efficacy of stroke therapies. The development and administration of biomaterials with appropriate physical properties to treat post-stroke inflammation is crucial; with additional complexities and potential advantages being acquired from the bioprinting of implantable tissues. Overall the vast array of NPs, hydrogels, and cell and gene therapies being investigated for the treatment of stroke is very promising and may lead to the licensing of a regenerative medicine inspired treatment in the years to come.

## Author contributions

OR and GP formulated original idea. EP contributed to the design of the review. OR, GP, and EP wrote, reviewed and approved the manuscript. GP designed the figures.

### Conflict of interest statement

The authors declare that the research was conducted in the absence of any commercial or financial relationships that could be construed as a potential conflict of interest.
